# Supplementary figures and images for: Transcriptional and Epigenomic Markers of the Arterial-Venous and Micro/Macro-Vascular Endothelial Heterogeneity within the Umbilical-Placental Bed
Source: Int J Mol Sci. 2022 Oct 6;23(19):11873. doi: 10.3390/ijms231911873 (PMC9569907; doi:10.3390/ijms231911873)

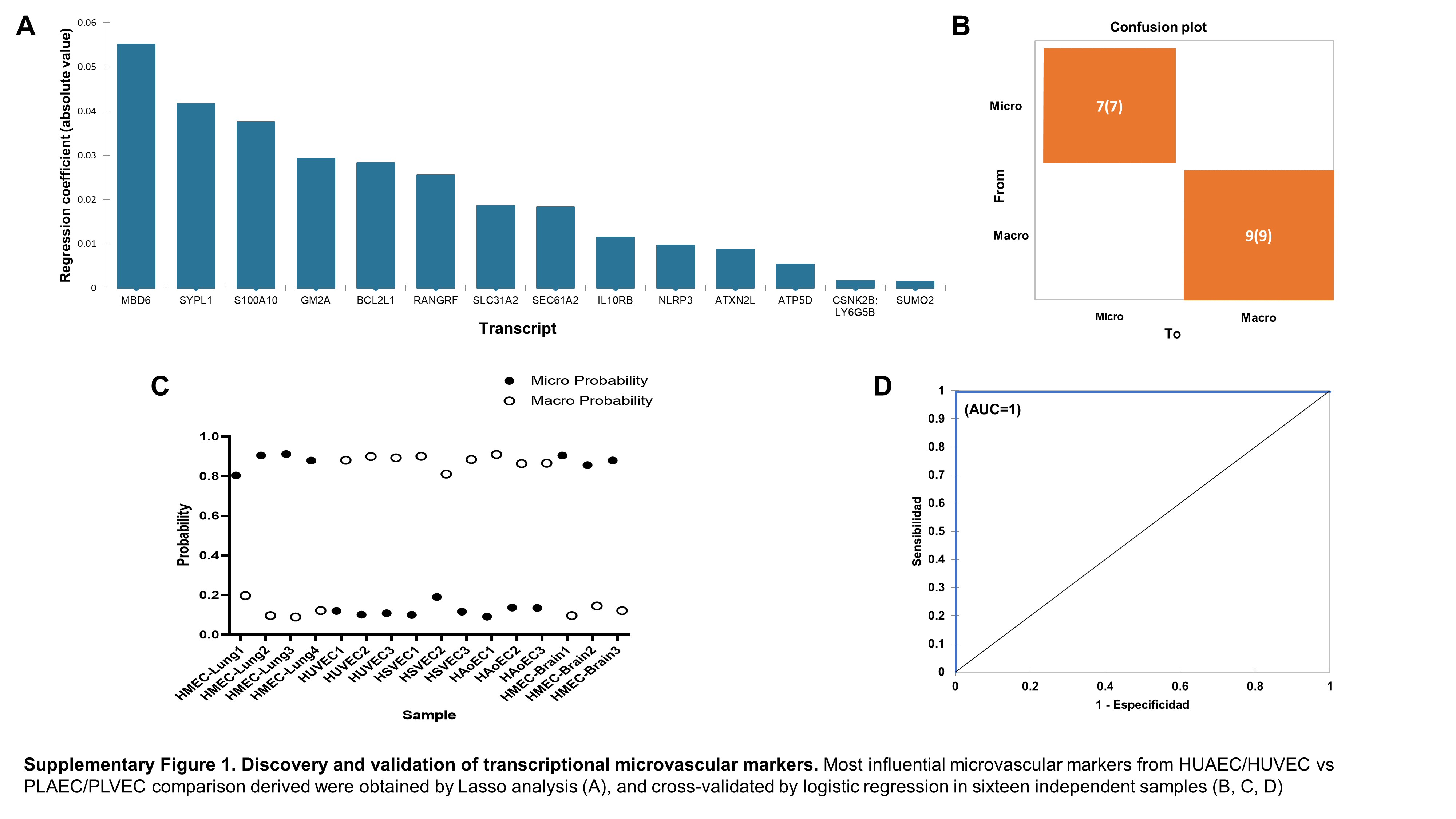

Supplement: Supplementary file 1 [file ijms-23-11873-s001.zip › Supplementary Figure S1.tif]
